# Copepod Lipidomics: Fatty Acid Substituents of Structural Lipids in *Labidocerca aestiva*, a Dominant Species in the Food Chain of the Apalachicola Estuary of the Gulf of Mexico

**DOI:** 10.3390/life15010043

**Published:** 2024-12-31

**Authors:** Paul L. Wood, Stan C. Kunigelis

**Affiliations:** 1 Metabolomics Unit, College of Veterinary Medicine, Lincoln Memorial University, 6965 Cumberland Gap Pkwy, Harrogate, TN 37752, USA; 2 Imaging and Analysis Center, DeBusk College of Osteopathic Medicine, Lincoln Memorial University, 6965 Cumberland Gap Pkwy, Harrogate, TN 37752, USA; stan.kunigelis@lmunet.edu

**Keywords:** copepods, lipidomics, *Labidocerca aestiva Wheeler*, *1900*, oceanic food chain, climate change, furan fatty acids, lipid remodeling

## Abstract

Zooplanktonic copepods represent a major biological mass in the marine food chain that can be affected by climate change. Monitoring the health of this critical biomass is essential for increasing our understanding of the impact of environmental changes on marine environments. Since the lipidomes of marine organisms are known to adapt to alterations in pH, temperature, and availability of metabolic precursors, lipidomics is one technology that can be used for monitoring copepod adaptations. Among the key lipid parameters that can be monitored are the fatty acid substituents of glycerolipids and glycerophospholipids. We utilized high-resolution tandem mass spectrometry (≤2 ppm mass error) to characterize the fatty acid substituents of triacylglycerols, glycerophosphocholines, ceramides, and sphingomyelins of *Labidocerca aestiva*. This included monitoring for furan fatty acid substituents, a family of fatty acids unique to marine organisms. These data will contribute to establishing a lipid database of the fatty acid substituents of essential structural lipids. The key findings were that polyunsaturated fatty acids (PUFAs) were only major substituents in glycerophosphocholines with 36 to 44 carbons. Triacylglycerols, ceramides, and sphingomyelins contained minimal PUFA substituents. Furan fatty acids were limited to mono- and di-acylglycerols. In summary, we have built a baseline database of the fatty acid substituents of key structural lipids in *Labidocerca aestiva*. With this database, we will next evaluate potential seasonal changes in these lipid substituents and long-term effects of climate change.

## 1. Introduction

**Figure 1:**
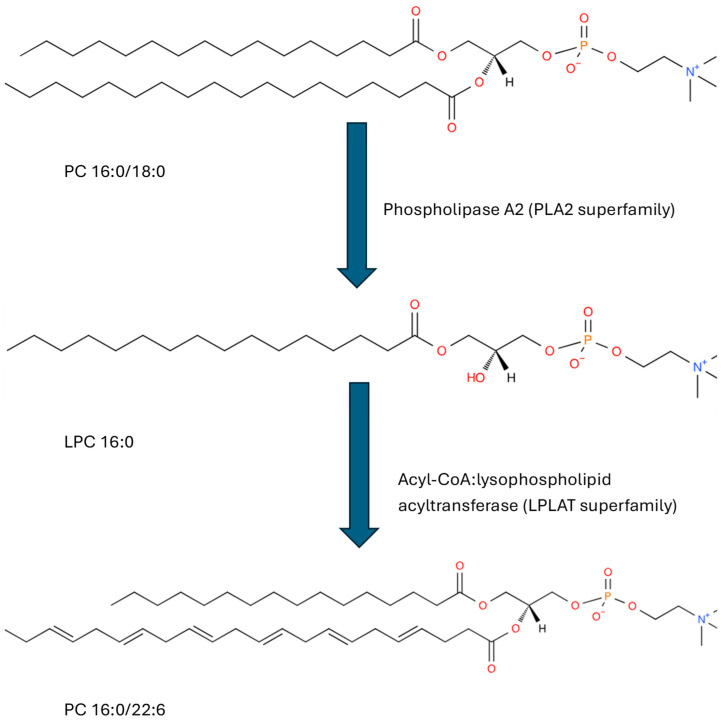
An example of glycerophosphocholine remodeling. PC 16:0/18:0 has fatty acid (FA) 16:0 at the sn-1 carbon of the glycerol backbone, FA 18:0 at sn-2, and the phosphocholine headgroup at sn-3. PLA2 enzymes remove the FA at sn-2, generating the lyso-lipid LPC 16:0, which is re-acylated with the PUFA 22:6 (DHA) via LPLAT enzymes.

It is important to monitor adaptations in the oceanic food chain to the dynamics of climate change, Lipidomics provides an in-depth evaluation of essential structural lipids. High-resolution mass spectrometry (HRMS) provides both the sensitivity and specificity required to monitor a diversity of lipids. Our lipid analytical platform monitors over 30,000 lipids across 195 lipid families [1,2] and has been utilized in a pilot study of copepod lipidomics [3]. Furthermore, identifying the fatty acid substituents in structural lipids supplies information on the availability of lipid precursors, including free fatty acid lipid remodeling [4]. Lipid remodeling of glycerolipids and glycerophospholipids mainly involves de-acylation via phospholipase A2 [5] and re-acylation via acyl-CoA: lysophospholipid acyltransferase [6], at sn-2 of the glycerol backbone (Figure 1), a major storage site for polyunsaturated fatty acids (PUFAs).

Lipid remodeling is key in providing PUFAs for cell signaling roles and in stabilizing membrane thickness and fluidity. De-acylation of saturated fatty acids and re-acylation with PUFAs decreases membrane viscosity, while re-acylation with longer-chain fatty acids increases membrane thickness, altering membrane packing and protein function [4]. In addition to saturated, unsaturated, and polyunsaturated fatty acids, many marine organisms synthesize furan fatty acids, which are incorporated into larger structural lipids [7,8]. These unusual fatty acids remain to be investigated in copepods.

Defining seasonal and climate-dependent lipid alterations in copepods is our long-term research goal. *Labidocerca aestiva* (*L. aestiva*), a dominant species in the food chain of the Apalachicola estuary of the Gulf of Mexico, represents a valuable copepod species for such studies. The long-term goal of our research is to evaluate the effects of climate change on the lipidome of copepods [4]. Establishing a database of the fatty acid substituents of glycerolipids, glycerophospholipids, and sphingolipids is important since alterations in these constituents, via lipid remodeling, are reflective of metabolic adaptations to environmental changes, as has been reported for bacteria [4] and for microalgae [9,10]. HRMS analysis of structural lipids provides valuable data on membrane and organelle structure that measurements of free fatty acids do not provide since free fatty acid levels are very dynamic and change within short timescales, while glycerolipids, glycerophospholipids, and sphingolipids are much more stable lipid pools. An analogy is the value of glycated hemoglobin (A1c) measurements over blood glucose levels in monitoring diabetes [11].

HRMS provides the sensitivity and specificity to monitor the fatty acid substituents of structural lipids with less than 2 ppm mass error [1,2]. NMR possesses the specificity but not the sensitivity for these studies, while gas chromatography does not provide structural analysis of large non-volatile structural lipids [12].

Environmental conditions are known to affect lipid metabolism in copepods and diatoms, their major food source. Transcriptomics and free fatty acid analyses have shown that fatty acid metabolism is down-regulated with increasing water temperatures [13,14]. This can have strong negative effects on the lipid accumulation required for diapause in copepods [15,16]. Other factors that impact lipid composition of copepod structural lipids involve available food supply and predation risks [17]. It is important to note that analysis of free fatty acids does not provide information on the structures of complex structural lipids. Our study is the first to define the fatty acid constituents of glycerolipids, glycerophospholipids, and sphingolipids in *L. aestiva.*

## 2. Materials and Methods

### 2.1. Copepods

*L. aestiva Wheeler. 1900* samples were harvested via netting from the Gulf of Mexico in May of 2024. Sea water was filtered in a plankton net with a 200 μm mesh, and the adult copepods (Stage 6) were identified and separated under 400× magnification light microscopy. Copepods were washed in distilled water and frozen at −20 °C for future analysis.

### 2.2. Lipidomics [1,2,3]

The cited references [1,2,3] are the foundation for the methods utilized in this study. Reference [2] is a detailed operating procedure providing all experimental details and sources of supplies and equipment.

The copepod samples (50 per analysis) were sonicated in 2 mL of methanol:water (1:1). Next, 2 mL of methyl-tert-butyl ether was added prior to vigorous shaking at room temperature for 30 min. To attain phase separation, the samples were centrifugated at 4000× *g* for 30 min. at room temp., and 1 mL of the upper organic layer was isolated and dried by centrifugal vacuum evaporation.

To reduce potential ion suppression, neutral and charged lipid species were separated prior to mass spectrometric analysis. Thermo HyperSep Cyano SPE cartridges (Thermo-Fisher, Waltham, MA, USA; 03-150-659) were conditioned with 5 mL of methyl-tert-butyl ether (MTBE)/chloroform (CHCl_3_)/acetic acid (98/2/0.2). The dried extracts were loaded onto the cartridge with the same conditioning solvent, and the neutral lipids eluted with 5 mL MTBE/CHCl_3_/acetic acid (98/2/0.2) followed by 5 mL of MTBE. The eluent was pooled and dried by vacuum centrifugation (Neutral Lipid Fraction). The charged lipids were eluted with 5 mL of MTBE/CHCl_3_/acetic acid (50/20/30) and dried by vacuum centrifugation.

The dried organic extracts were dissolved in infusion solution (2-propanol:methanol:chloroform:H_2_O (160:80:80:1), with the water containing 15 mg of NH_4_Cl) for mass spectrometric analyses. For flow infusion analysis, the samples were infused at 12 μL per min into the ESI source for high-resolution data acquisition (140,000, <2 ppm mass error), with an orbitrap mass spectrometer (Thermo Q Exactive, Waltham, MA, USA). The [M + H]^+^ ions were monitored for glycerophosphocholines (PC), lysophosphocholines (LPC), ceramides, and sphingomyelins (SM). The [M + NH_4_]^+^ ions were monitored for triacylglycerols (TG), furan diacylglycerols, and furan monoacylglycerols. Between injections, the transfer line was washed with successive 1 mL washes of methanol and hexane/ethyl acetate/chloroform/H_2_O (3:2:1:0.1).

Structural validation was achieved via tandem mass analyses (MS^2^). For MS^2^ studies, parent ions were selected with a 0.4 amu window, and product ions (Table 1) monitored with 140,000 resolution (<2 ppm mass error). The HCD energies used were 15, 25, and 35 arbitrary units.

### 2.3. Data Analysis

Our analytical platform utilizes an Excel spreadsheet database of over 30,000 unique lipids, which includes exact masses and calculated ion adducts. Based on our infusion solvent, the predominant ions monitored were [M + H]^+^ or [M + NH_4_]^+^ in positive electrospray ionization (PESI), while they were [M-H]^−^ or [M + Cl]^−^ in NESI. The HRMS scan data were imported into this Excel spreadsheet. Scan data with less than 2 ppm mass error for the calculated ion adduct were recorded as hits, and the peak intensity for each hit imported into the spreadsheet [3]. For each lipid family, the family members were ranked, with the largest peak intensity ranked as 1.0. All data are the averages of 3 independent determinations.

### 2.4. Nomenclature

Lipid nomenclature adheres to the guidelines of Lipid Maps [18]. Carbon positions are counted from the terminal carboxy group. Lipid notation such as 20:5 indicates 20 carbons and 5 double bonds. Lyso is shorthand for loss of a fatty acid substitution.

## 3. Results

### 3.1. Glycerophosphocholines (GPC)

*L. aestiva* was found to possess a diversity of GPCs, ranging from GPC 28:0 to GPC 44:12 (Table 2; Supplementary Table S1). GPCs are the predominant GPL in copepods [3]. All GPCs were validated by the MS^2^ PESI product 184.0733. In addition, the chloride adducts of these lipids in NESI were utilized to determine the fatty acid substituents [1,2].

The higher molecular weight GPCs were rich in the PUFAs 22:6 (DHA, docosahexaenoic acid) > 20:5 (EPA, eicosapentaenoic acid) > 20:4 (AA, arachidonic acid) > 22:5 (DPA, docosapentaenoic acid) (Table 2), while saturated, mono-unsaturated, and di-unsaturated fatty acids were dominant in lower molecular weight GPCs (Table 2).

The same copepod lipid extracts also contained high levels of lysoglycerophosphocholines (LPCs) (Supplementary Table S1). Of interest, the LPCs containing PUFAs were 20:5, 22:5, and 22:6, but not 20:4. As with GPCs, LPCs were validated by the MS^2^ PESI product 184.0733, and fatty acids verified in NESI with MS^2^ of the chloride adducts. No furan fatty acid substituents were monitored for GPCs or LPCs.

In summary, the size of the GPC pool in copepods, along with the dynamic response of de-acylation/re-acylation remodeling to environmental changes, makes this lipid family a valuable target for lipidomics analyses of seasonal and climate-dependent alterations in the copepod lipidome.

### 3.2. Triglycerides (TGs)

TGs are the major constituents of lipid storage droplets in copepods [3]. Lipid droplets, which form in the endoplasmic reticulum, serve as reservoirs of neutral lipids, which are mainly TGs and sterol esters. These intracellular structures consist of a GPL monolayer around a neutral lipid core, serving roles in energy storage and the supply of fatty acids for lipid biosynthesis and cell signaling.

We observed that in contrast to PCs, the major fatty acid substituents of TGs in *L. aestiva* were saturated fatty acids, with only FA 20:5 as a PUFA substituent (Table 3; Supplementary Table S1). No furan fatty acid substituents were monitored for TGs.

### 3.3. Sphingolipids: Ceramides

Both deoxyceramides and ceramides were monitored in lipid extracts of *L. aestiva* (Table 4; Supplementary Table S1). We were the first to report high levels of deoxyceramides in copepods [3]. Our current data indicate that for deoxyceramides, the sphingolipid bases are m2 and m3, while the fatty acids are mainly saturated (Table 4). The sphingolipid bases for deoxyceramides were validated by tandem MS in PESI (Table 1), which generated the sole product ion of [MH-H_2_O]^+^. Deoxyceramides, which utilize alanine rather than serine as a precursor, have a reduced hydrogen bond capacity, making them more hydrophobic and less organized in membranes [19]. These ceramides appear to be involved in the regulation of autophagy [20].

In the case of canonical ceramides, the sphingolipid bases were found to be d18:1, d18:2, and d18:3, with saturated fatty acids as the dominant substituents. The sphingolipid bases for ceramides were validated by tandem MS in PESI (Table 1), which generated the product ions of [MH-H_2_O]^+^, [MH-2H_2_O]^+^, and [MH-C-2H_2_O]^+^ (Table 1). No furan fatty acid substituents were monitored for ceramides.

### 3.4. Sphingolipids: Sphingomyelins

Lipid extracts of *L. aestiva* (Table 5; Supplementary Table S1) contained a wide diversity of sphingomyelins spanning SM 32:2 to SM 44:2. Validation of sphingomyelins was obtained with the PESI product ion of phosphocholine [184.0733]^+^ using tandem MS (Table 1). A unique feature of copepods is their myelinated axons [21], which presumably possess the monitored sphingomyelins. No furan fatty acid substituents were monitored for sphingomyelins.

### 3.5. Furan Lipids

**Figure 2:**
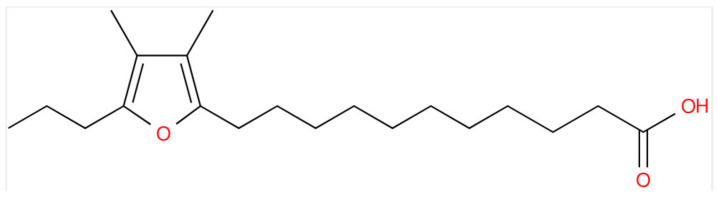
Structure of 11-(3,4-dimethyl-5-propylfuran-2-yl)-undecanoic acid [11-D-3; CAS 57818-41-4; LMFA01150012; 322.250795; LogP 6.7]. The universal abbreviations for furan fatty acids are number-letter-number notations, with the first number designating the length of the carboxyalkyl chain. The M or D designate 1 (M, mono) or 2 (D, di) methyl moieties on the furan ring, while the second letter designates the length of the alkyl chain.

A number of marine species synthesize furan fatty acids (Figure 2), which are present as fatty acid substituents in glycerolipids, glycerophospholipids, and cholesterol esters [7,8,22,23,24]. Cyclization to form the furan ring involves hydroperoxy fatty acids in algae and methylated fatty acids in bacteria [25].

In our lipid extracts of *L. aestiva*, we detected mono- and di-acylglycerols with furan fatty acid substituents (Table 6; Supplementary Table S1). The impact of these unusual fatty acids on membrane function is unknown at this time.

## 4. Discussion

Lipid remodeling, in response to developmental and environmental alterations, involves alterations in fatty acid substituents in glycerolipids, glycerophospholipids, and sphingolipids. In addition, this can involve alterations in the headgroups of glycerophospholipids based on nutrient supply [26,27]. Alterations in fatty acid substituents [28,29] and lipid headgroups [30] all can alter membrane fluidity and lipid raft function.

While there are numerous reports of free fatty acids in copepods [31], definitions of the fatty acid substituents of specific glycerophospholipids, glycerolipids, and sphingolipids have not been conducted. This is the first study to provide a database of these lipid substituents. A significant finding was that polyunsaturated fatty acids (PUFAs) are dominant in glycerophosphocholines, but not in triglycerides or ceramides. PUFAs are critical in maintaining membrane fluidity and, after release during lipid remodeling (Figure 1), serve as precursors to a number of cell-signaling molecules involved in inflammatory cascades and in the resolution of these processes [32,33]. They include prostaglandins and oxylipins, which have been characterized in copepods [3,32,34]. Prostaglandins and oxylipins have also been monitored in microalgae [3,34]. In this regard, oxylipin aldehydes secreted by microalgae are used to deter grazing by copepods [35].

Lipid droplets contain the major cellular stores of TGs and cholesterol esters in copepods [36]. We were surprised to find that these TG stores were devoid of PUFAs since we previously monitored cholesterol ester PUFA substituents in copepods [3]. This finding may relate to the observations that copepods contain all of the enzyme machinery required to synthesize PUFAs [37], providing independence from dietary PUFAs.

The occurrence of furan fatty substituents of glycerolipids and glycerophospholipids has been reported for a number of marine organisms [25]; however, ours is the first report of furan fatty acid substituents for MGs and DGs in copepods. The functional impact of these unusual lipids on membrane structure and cellular metabolism is unknown at this time, except for a conjectured antioxidant role. Also, we do not know whether these furan lipids were ingested and/or synthesized by copepods.

## 5. Conclusions

The precision and sensitivity of our lipidomics analytical platform, along with our database of fatty acid substituents of structural lipids, will allow for quantitation of seasonal and climate-dependent alterations in the lipidome of *L. aestiva*. Monitoring alterations in fatty acid substituents will provide a sensitive measure of the effects of environmental stressors on copepod populations, which are essential in the oceanic food chain.

The major findings of our study were the importance of glycerophospholipid pools for the storage of PUFAs and the first report of furan fatty acid substituents in copepods.

Study Limitations: *Biological:* Copepods have complex life cycles, and their lipidomes will vary with those cycles and the seasons. We have only taken a snapshot of one point in time with this probe study. Larger sample sets from different seasons of the year will be crucial to build a lipid database for a long-term climate change study.

Study Limitations: *Technical:* Our HR-MS analytical platform (<2 ppm mass error), which utilizes both PESI and NESI, significantly reduces the risk of lipid misassignments.

## Figures and Tables

**Table 1:** Product ions for validation of monitored lipid families.

Lipid Family	Precursor	Product	[Product Ion]^+^
PC and LPC	[M + H]^+^	Phosphocholine	[184.0733]^+^
SM	[M + H]^+^	Phosphocholine	[184.0733]^+^
Cer	[M + H]^+^	Sphingosine base: [M-H_2_O, M-2H_2_O, M-C-2H_2_O]^+^	[d17:0 = 252.2685/270.2791/240.2685]^+^ [d18:1 = 264.2685/282.2791/252.2685]^+^ [d18:2 = 262.2529/280.2634/250.2529]^+^ [d18:3 = 260.2372/278.2478/248.2372]^+^
DeoxyCer	[M + H]^+^	Sphingosine base: [M-H_2_O]^+^	[m16:0 = 240.26858]^+^ [m17:0 = 254.28423]^+^ [m18:2 = 264.26858]^+^ [m18:3 = 262.25293]^+^
TAG	[M + NH_4_]^+^	[MH-FA]^+^	[MH-FA]^+^
Furan MG/DG	[M + NH_4_]^+^	[Furan FA – H_2_O]^+^	[Furan FA 18:0 = 277.2162]^+^ [Furan FA 20:0 = 305.2475]^+^ [Furan FA 22:0 = 333.2788]^+^

**Table 2:** Rank order and fatty acid substituents of glycerophosphocholines (GPC) in *L. aestiva.* The dominant PC (i.e., Rank = 1.0) is highlighted in orange.

GPC	Rank Order [Fatty Acid Composition]
PC 28:0	0.2834 [14:0/14:0]
PC 30:0	0.8302 [14:0/16:0]
PC 30:1	0.2479 [16:1/14:0]
PC 32:0	0.5876 [16:0/16:0]
PC 32:1	0.2457 [16:0/16:1, 14:0/18:1]
PC 32:2	0.1428 [14:0/18:2, 16:1/16:1]
PC 32:4	0.04096 [18:4/14:0]
PC 33:0	0.0681 [17:0/16:0]
PC 33:1	0.0481 [17:1/16:0, 15:0/18:1]
PC 33:5	0.12138
PC 33:6	0.03434
PC 34:0	0.0394 [18:0/16:0]
PC 34:1	0.0920 [18:1/16:0]
PC 34:2	0.0943 [16:0/18:2]
PC 34:3	0.16972
PC 34:4	0.1111 [16:0/18:4]
PC 34:5	0.1526 [16:1/18:4]
PC 34:6	0.02901
PC 36:2	0.0154 [18:1/18:1]
PC 36:3	0.03066 [20:3/16:0]
PC 36:4	0.10067 [20:4/16:0]
PC 36:5	0.5466 [20:5/16:0]
PC 36:6	0.5033 [20:5/16:1, 14:0/22:6]
PC 38:4	0.02938 [18:0/20:4]
PC 38:6	1.0 [16:0/22:6]
PC 38:7	0.2257 [18:2/20:5, 16:1/22:6]
PC 40:6	0.1718 [18:0/22:6]
PC 40:7	0.0995 [18:1/22:6, 20:2/20:5]
PC 40:8	0.0955 [20:4/20:4, 18:2/22:6]
PC 40:10	0.2213 [20:5/20:5, 18:4/22:6]
PC 42:10	0.1316 [20:4/22:6, 20:5,/22:5]
PC 42:11	0.5727 [20:5/22:6]
PC 44:12	0.3802 [22:6/22:6]

**Table 3:** Rank order and fatty acid substituents of triacylglycerols (TG) in *L. aestiva.* The dominant TG (i.e., Rank = 1.0) is highlighted in orange.

Triacylglycerol (TG)	Rank Order [Fatty Acid Composition]
TG 45:0	0.1019 [14:0/16:0/15:0]
TG 45:1	0.05555
TG 46:0	0.3744 [16:0/16:0/14:0 & 14:0/14:0/18:0]
TG 46:1	0.5269 [16:1/16:0/14:0]
TG 46:2	0.2258 [16:1/16:1/14:0]
TG 47:0	0.183 [16:0/16:0/15:0 & 16:0/17:0/14:0]
TG 47:1	0.2231 [16:1/16:0/15:0]
TG 47:2	0.07251
TG 48:0	0.2911 [16:0/16:0/16:0 & 16:0/18:0/14:0]
TG 48:1	0.6533 [16:0/16:0/16:1 & 16:0/14:0/18:1]
TG 48:2	0.5594 [16:0/16:1/16:1]
TG 50:1	0.4031 [18:1/16:0/16:0 & 16:1/18:0/16:0]
TG 50:2	0.4339 [18:1/18:1/14:0 & 18:1/16:1/16:0]
TG 50:3	0.3149 [16:0/16:1/18:2 & 16:0/16:0/18:3]
TG 50:4	0.33276
TG 50:5	0.57729
TG 51:1	0.03143
TG 51:2	0.02808
TG 51:3	0.03548
TG 51:4	0.04182
TG 51:5	0.17014
TG 51:6	0.13825
TG 52:0	0.09811 [18:0/18:0/16:0 & 20:0/16:0/16:0]
TG 52:1	0.10645
TG 52:2	0.18845
TG 52:3	0.1653 [18:2/18:1/16:0]
TG 52:4	0.2252 [18:2/18:2/16:0]
TG 52:5	0.64034 [20:5/16:0/16:0]
TG 52:6	0.7619
TG 52:7	0.33678
TG 54:3	0.08501 [18:1/18:1/18:1]
TG 54:5	0.3683 [18:2/18:2/18:1]
TG 54:6	1.0 [18:2/18:2/18:2 & 20:5/18:1/16:0]
TG 54:7	0.64591
TG 54:8	0.30297
TG 56:6	0.40651
TG 56:7	0.38352
TG 56:8	0.22942
TG 56:9	0.27218
TG 56:10	0.44847
TG 56:11	0.26247
TG 58:6	0.06022
TG 58:10	0.31871
TG 58:11	0.71405

**Table 4:** Rank order and fatty acid substituents of ceramides in *L. aestiva.* The dominant Cer (i.e., Rank = 1.0) is highlighted in orange.

Deoxyceramides	Rank Order [Fatty Acid Composition]
Cer 34:2; O	0.3896 [m18:2/16:0]
Cer 34:3; O	1.0 [m18:3/16:0]
Cer 36:3; O	0.2347 [m18:3/18:0]
Cer 42:3; O	0.2286 [m18:2/24:1, m18:3/24:0
Cer 42:4; O	0.9685 [m18:3/24:1]
Cer 44:4; O	0.1362 [m18:2/26:2, m17:0/27:4]
Ceramides	Rank Order [Fatty Acid Composition]
Cer 34:2; O2	0.3090 [d18:1/16:1, d17:0/17:2]
Cer 36:2; O2	0.0694 [d18:2/18:0]
Cer 36:3; O2	0.0669 [d18:3/18:0]
Cer 40:3; O2	0.1032 [d18:3/22:0]
Cer 42:2; O2	0.3391 [d18:1/24:1. d18:2/24:0]
Cer 42:3; O2	1.0 [d18:2/24:1, d18:3/24:0]
Cer 42:4; O2	0.20808 [d18:3/24:1]
Cer 44:3; O2	0.2875 [d18:2/26:1, d18:3/26:0]
Cer 44:4; O2	0.1381 [d18:3/26:1, d18:2/26:2]

**Table 5:** Rank order of sphingomyelins in *L. aestiva.* The dominant SM (i.e., Rank = 1.0) is highlighted in orange.

Sphingomyelin (SM)	Rank Order
SM 32:2;O2	0.02089
SM 34:1;O2	0.04334
SM 34:2;O2	0.23157
SM 34:3;O2	0.07584
SM 35:3;O2	0.43878
SM 36:3;O2	0.05708
SM 37:3;O2	0.34683
SM 38:3;O2	0.02668
SM 39:3;O2	0.04634
SM 40:2;O2	0.03321
SM 40:3;O2	0.0712
SM 40:4:O2	0.02315
SM 41:3;O2	0.17272
SM 41:4:O2	0.21286
SM 42:2;O2	0.16991
SM 42:3;O2	0.44903
SM 42:4:O2	0.10262
SM 43:3;O2	0.16701
SM 43:4:O2	1.0
SM 44:3;O2	0.25774
SM 44:4:O2	0.09139

**Table 6:** Rank order of furan lipids in *L. aestiva.* The dominant furan DG and furan MG (i.e., Rank = 1.0) are highlighted in orange.

Furan Diacylglycerol	Rank Order [Fatty Acid Composition]
Furan DG 34:0	1.0 [Furan FA 18:0 = 277.2162; 0.20 ppm]+
Furan DG 36:0	0.6698 [Furan FA 18:0 = 277.2162; 0.22 ppm]+
Furan DG 38:0	0.514 [Furan FA 18:0 = 277.2162; 1.4 ppm]+
Furan DG 40:0	0.482 [Furan FA 22:0 = 333.2788; 0.30 ppm]+
Monoacylglycerol Furan	Rank Order [Fatty Acid Composition]
Furan MG 20:0	1.0 [Furan FA 20:0 = 305.2475; 1.4 ppm]+
Furan MG 22:0	0.1558 [Furan FA 22:0 = 333.2788; 0.63 ppm]+

## Data Availability

All data are included in the manuscript and Supplementary Table S1.

## Supplementary Materials

The following supporting information can be downloaded at: https://www.mdpi.com/article/10.3390/life15010043/s1, Table S1: GPCs, ceramides, SM, carotenoids, TAG, furan lipids.
